# Engineered Multilayer Microcapsules Based on Polysaccharides Nanomaterials

**DOI:** 10.3390/molecules25194420

**Published:** 2020-09-25

**Authors:** Salvatore Lombardo, Ana Villares

**Affiliations:** INRAE, UR BIA, F-44316 Nantes, France; ana.villares@inrae.fr

**Keywords:** nanocellulose, cellulose nanofibers, cellulose nanocrystals, layer-by-layer, coating, microencapsulation, core responsive

## Abstract

The preparation of microcapsules composed by natural materials have received great attention, as they represent promising systems for the fabrication of micro-containers for controlled loading and release of active compounds, and for other applications. Using polysaccharides as the main materials is receiving increasing interest, as they constitute the main components of the plant cell wall, which represent an ideal platform to mimic for creating biocompatible systems with specific responsive properties. Several researchers have recently described methods for the preparation of microcapsules with various sizes and properties using cell wall polysaccharide nanomaterials. Researchers have focused mostly in using cellulose nanomaterials as structural components in a bio-mimetic approach, as cellulose constitutes the main structural component of the plant cell wall. In this review, we describe the microcapsules systems presented in the literature, focusing on the works where polysaccharide nanomaterials were used as the main structural components. We present the methods and the principles behind the preparation of these systems, and the interactions involved in stabilizing the structures. We show the specific and stimuli-responsive properties of the reported microcapsules, and we describe how these characteristics can be exploited for specific applications.

## 1. Introduction

Microcapsules can be defined as particles with micrometer dimensions that can be used to store compounds inside them [1]. The process used to entrap small particles of liquids, solids, or gases in the hollow cavity of microcapsules is called microencapsulation [2]. The preparation of microcapsules using materials based on natural resources is currently receiving great attention, and has been the focus of recent reviews [3,4]. Such systems have great potential in food science and for the development of novel drug-delivery systems, since they can provide protection against degradation and an improved release profile of the encapsulated species [5,6]. Microcapsules have been exploited in several other applications, such as textiles [7], cosmetics [8], agriculture [9], construction [10], and printing [11]. Microcapsules are formed by the deposition of a coating material onto a sacrificial template. Then, a cavity is formed by removal of the template. Coating can be stabilized by covalent bonds or weaker interactions [12]. The active substance can be encapsulated by direct coating, or by preloading in a porous template, which is dissolved in a successive step. [13] For applications such as drug or food delivery, the use of biocompatible polymers as coating materials is crucial. For this purpose, polymers obtained from natural monomers, such as lactic or glycolic acid, have been used to prepare microcapsules, and have been focus of recent reviews [14,15,16]. The use of natural polymers like polysaccharides for the preparation of microcapsules is of particular interest, because of their large availability and their biocompatibility. Several systems have been presented in literature, and the interactions involved in stabilizing the microcapsule structure have been described [12,17]. Examples of preparation of hollow capsules from cellulose fibers with diameter ranging from ≈ 30 µm to few mm have been reported [18,19]. However, polymeric cellulose esters have been more commonly used as coating materials for drug delivery applications [20,21]. One of the most used natural polysaccharide to prepare microcapsules in the pharmaceutical industry is chitosan. Various techniques to prepare chitosan microcapsules for encapsulation of drugs have been presented [22] Alginate is another natural polysaccharide that has been widely used to prepare microcapsules with great potential as drug delivery carrier, [23,24] or for encapsulation of oils [25]. Among other polysaccharides, starch has been largely used to prepare microcapsules for food applications[26]. Recently, the scientific community has shown increasing interest in using polysaccharide nanoparticles as coating materials for the preparation of microcapsules. Polysaccharide nanomaterials can be extracted from many natural resources. Their extraction may represent a promising way for recycling organic waste [27,28,29]. An advantage of using highly crystalline polysaccharide nanomaterials is linked to their high stiffness and tensile strength [30,31]. In particular, cellulose and chitin nanocrystals are widely used as reinforcing agents, [32,33] and their characteristics could be exploited to prepare microcapsules with improved mechanical properties. In particular, the use of cellulose nanomaterials as main coating component has attracted much interest, because of the large availability of cellulose in nature, and because they can be extracted using less harsh conditions, compared to other polysaccharide nanomaterials [34]. As cellulose is one of the main components of the plant cell wall, [35] cellulose nanomaterials could be used in a biomimetic approach to prepare microcontainers inspired by nature. Cellulose nanomaterials have low toxicity, [36] and nanocellulose-loaded microcapsules would represent a promising platform for the development of novel drug-delivery systems and other biomedical applications, such as biosensors. A biosensor is formed by a biologically active element immobilized on a convenient substrate (such as a cellulose nanomaterial), a transducer, and a signal processor. A common challenge in the preparation of biosensors is linked to the immobilization of the biological active materials on the substrate [37]. An example of an engineered biosensor based on nanocellulose was presented by Khattab et al., where the authors immobilized the enzyme urease onto a film prepared by cellulose nanocrystals and a spectroscopic probe, to monitor the urea concentration [38]. Encapsulation of active molecules could represent an alternative to immobilization on a film, and nanocellulose-based microcapsules represent a promising platform for developing novel biosensors. In addition, the surface reactivity of cellulose nanoparticles could be exploited to directly graft the active molecule in the microcapsules shell. Cellulose nanomaterials are promising building blocks to prepare three-dimensional structures, and they are very attractive for various other biomedical applications, such as wound healing. Various examples of recently investigated advanced nanocellulose materials for biomedical applications were described in a recent review [39]. For instance, Liu et al. reported the preparation of hydrogels prepared combining cellulose nanofibers and hemicelluloses for wound healing applications [40]. Inserting microcapsules in such three-dimensional materials could be particularly advantageous for wound healing application, as it would enable to insert specific drugs by encapsulation, for example to provide anti-inflammatory properties. An additional advantage of using cellulose nanomaterials to create three-dimensional structures is their printability, for example using the direct ink writing technique, which enables programmable assembly of three-dimensional periodic architectures [41]. Among other crystalline polysaccharide nanomaterials, chitin and starch nanoparticles have also been used as coating for microcapsules. In this review, we will briefly describe polysaccharide nanomaterials, and show the main methodologies employed to prepare microcapsules with polysaccharide nanomaterials as coating component. We will review most of the recent work published, with particular focus on the architectures reported, on the interactions used to stabilize the structure, and on the specific properties of the reported microcapsules.

## 2. Polysaccharide Nanomaterials

**Figure 1:**
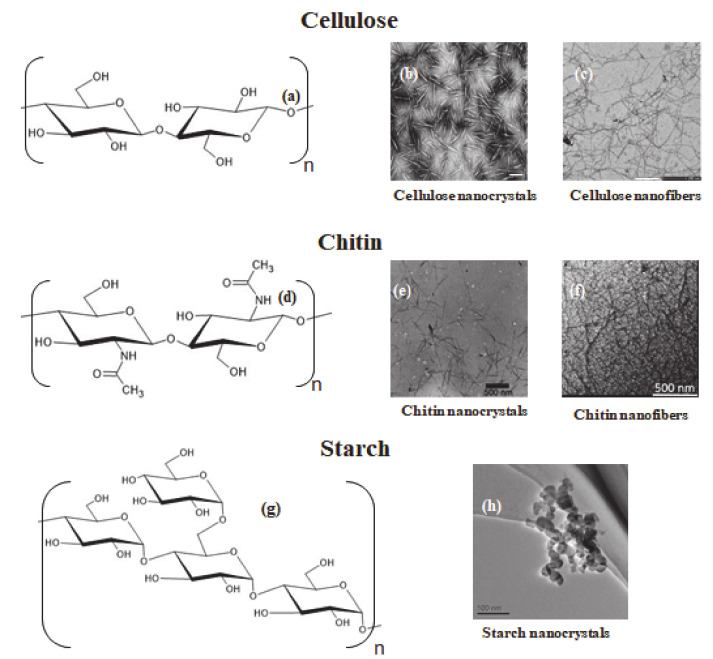
Repeating unit structures for cellulose, chitin, and starch, and TEM micrographs of their corresponding nanoparticles. (**a**) Repeating unit of cellulose. (**b**) TEM micrograph of cellulose nanocrystals, reproduced from Ref. [43] with permission from The Royal Society of Chemistry, scale 200 nm. (**c**) TEM micrograph of cellulose nanofibers, reproduced from Ref. [44] with permission from The Royal Society of Chemistry, scale 200 nm. (**d**) Repeating unit of chitin. (**e**) TEM micrograph of chitin nanocrystals, reprinted with permission from [45]. Copyright (2007) American Chemical Society. (**f**) TEM micrograph of chitin nanofibers, reprinted with permission from [46]. (**g**) Repeating unit of starch. (**h**) TEM micrograph of starch nanocrystals, reprinted with permission from [47] Copyright © 2010 Published by Elsevier Ltd.

Polysaccharides are polymers widely available in nature, composed of monosaccharide units bound by glycosidic bonds. Several polysaccharides have been used to prepare nanomaterials, and several preparation routes have been presented [42]. Here, we will focus more specifically on the polysaccharides nanomaterials used to prepare microcapsules, namely cellulose, chitin, and starch. An overview of their structure is presented in Figure 1.

### 2.1. Cellulose Nanomaterials

Cellulose is the most abundant naturally occurring biopolymer in the world. It is normally extracted from plants, and constitutes the main structural component of the plant primary cell wall [34]. Cellulose fibers are also produced by other eukaryotic species, such as oomycotes [48], algae [49], and tunicates [50]. Some species of bacteria are also known to secret cellulose [51]. Cellulose is a homopolysaccharide composed by repeating β-D-anhydroglucopyranose units, linked by β(1 → 4) glycosidic bonds [52]. The structure of the repeating unit of cellulose is shown in Figure 1a. Cellulose nanofibers (CNF) can be extracted using various physical and chemical methods. Generally, cellulose fibers are disintegrated into fine fragments, or cellulose nanofibers, by mechanical refining methods. The high energy consumption of these procedures requires the pretreatment of cellulose fibers by enzymes or chemicals. The resultant CNF consist of microfibrils with diameters of a few nanometers, and lengths in the micrometer range (see Figure 1b) [53]. Cellulose nanocrystals (CNC) are obtained by hydrolysis performed with a strong acid, which preferentially hydrolyzes the disordered regions of cellulose leaving intact the crystalline region [54]. The resulting cellulose rod-like nanoparticles have a width of 3–30 nm and a length of 70–1000 nm, depending on the source and hydrolysis conditions (see Figure 1c) [55]. The structure of cellulose nanocrystals and nanofibers, characterized by TEM, is shown in Figure 1b, c, respectively. Sulfuric and hydrochloric acid are normally used to prepare cellulose nanocrystals, resulting in negatively charged and neutral CNCs, respectively [56]. The surface of cellulose nanomaterials can be functionalized through chemical reactions. TEMPO ((2,2,6,6-Tetramethylpiperidin-1-yl)oxyl) oxidation is one of the most commonly used modification, as it has the advantage to selectively oxidize the primary hydroxyl groups resulting to carboxylated nanocellulose [57]. The reactivity of the hydroxyl groups on nanocellulose surface gives many possibilities of chemical modification, and this topic was the focus of recent reviews [52,58].

### 2.2. Chitin Nanomaterials

Chitin is the second most abundant biopolymer in the world, after cellulose [59]. It can be extracted from shellfishes, but also from other biological sources, such as worms, insects, or fungi [60]. Chitin is composed by repeating *N*-acetylglucosamine units linked by β(1→4) glycoside bonds [60]. The structure of the repeating unit of chitin is shown in Figure 1d. Preparation of rod-like chitin nanocrystals (ChiNC) was firstly reported in 1959, from hydrochloric acid hydrolysis of crab shell [61]. Various sources and methods have been proposed for the preparation of chitin nanofibers and nanocrystals, with size being dependent on the preparation method and sources [62]. The structure of chitin nanocrystals and nanofibers is related to that of cellulose nanomaterials (see Figure 1e,f), but they have opposite charge, due to the surface amino groups. Due to the repulsion forces between positive charges, chitin nanocrystals form stable suspensions in water [63]. However, the surface charge can be modulated by the degree of acetylation and through surface modifications, [62] including TEMPO-mediated oxidation [64]. Preparation of spherical chitin nanoparticles has also been reported [65,66]. Chitosan is a polymer derived by the partial *N*-deacetylation of chitin, widely available in nature. Several preparation methods of chitosan nanoparticles have been presented, and have been the subject of recent reviews [67,68].

### 2.3. Starch Nanomaterials

Starch is a biopolymer produced by plants as an energy storage source, and it has always been used by humans as food source and to prepare materials [69]. Starch is composed of two main structural components, amylose and amylopectin. Amylose is a linear polysaccharide constituted of glucose units linked by α-1,4 glycosidic bonds. Amylopectin differs with amylose for the presence of branches, with an α-1,6 linkage every 24–30 glucose units [70]. The structure of the repeating unit of starch is shown in Figure 1g. Starch is found as microgranules with dimensions dependent on the botanical sources, which can range from few µm (such as rice starch) to more than 100 µm (such as potato starch) [71]. Starch nanocrystals (SNC) are obtained by acid hydrolysis of starch granules, which is usually performed with sulfuric acid, [72] or with hydrochloric acid [73]. Hydrolysis of starch with sulfuric acid leads to negatively charged nanocrystals and present better colloidal stability than nanocrystals prepared from hydrochloric acid [74]. The morphology and the size of starch nanocrystals depends on the sources and hydrolysis conditions [75]. For instance, hydrolysis with sulfuric acid resulted to platelet-like structures (see Figure 1h) with length ranging around 25–120 nm [76]. Non-crystalline starch nanoparticles have also been prepared by mechanical treatments or from regeneration of starch solution in organic solvents [77].

## 3. Methods of Microcapsule Fabrication

Microcapsules are normally prepared by coating a core material, which acts as template, with a polymer film. In this section, we will review the main methods available for polysaccharide nanoparticles as coating material. The core materials used to prepare microcapsules from polysaccharide nanomaterials were either solid or liquid. Core materials have been classified according to their phase, as “hard template” if the core is solid, or “soft template” if the core is a liquid.

Hard templates can be coated using any molecular interaction between the solid core and the polysaccharide nanomaterial. Interactions between polysaccharides and other molecules are dependent on different contributions, and interactions on nanocellulose surfaces have been described in a recent review [78]. One can assume a comparable behavior between other polysaccharide nanoparticles and other molecules, considering that polysaccharides form hydrogen bonds by the surface hydroxyl groups, and they possess surface charge, which can be used to direct electrostatic interactions with species of opposite charge. The hard template can be dissolved in a further step, to yield hollow porous microcapsules. Soft templates have also been extensively used as core material to prepare microcapsules. For example, the method of interfacial polymerization has been described,[1] and used to prepare microcapsules with polyamide-polyurea as hybrid shell [79]. The use of Pickering emulsion, which are emulsions stabilized by solid particles, [80] can be particularly interesting, as the interaction between the solid particles and the liquid droplets can be exploited to encapsulate liquids in a single step, or to prepare liquid core microcapsules [81,82]. The use of soft template is particularly useful for polysaccharide nanomaterials due to their amphiphilic properties, which can be exploited to stabilize Pickering emulsions. Particles with high aspect ratio, such as rod-shaped cellulose nanomaterials and other polysaccharides, are advantageous for Pickering emulsion stabilization, since they require lower concentration compared to spherical particles [83]. The behavior of polysaccharide nanomaterials at the interphase has been subject of recent reviews [67,84,85]. Oza and Frank reported the earlier scientific studies that demonstrated the ability of microcrystalline cellulose to stabilize oil-in-water (*o*/*w*) and multiple (*w*/*o*/*w*) emulsions. The authors explained the mechanism showing that the cellulose fibers adsorbed on the oil–water interface, forming a network that acted as a mechanical barrier against coalescence [86,87]. Pickering emulsions stabilized by bacterial cellulose nanofibers presented higher stability, which was associated with the smaller size of bacterial cellulose fibrils, which were able to form a scaffolding structures around the oil droplet in addition to the network structure [88]. More recently, Capron’s group used smaller cellulose nanocrystals with low surface charge to prepare stable oil-in-water Pickering emulsions by sonication, using hexadecane as the oil phase. The authors showed a decrease in droplet size at higher CNC concentration, associated with an increase of repulsion forces between CNCs, which would destabilize the emulsion, [89] and that a very low surface charge density was required to prepare stable emulsions [90]. Cellulose nanocrystals formed a monolayer around the emulsion droplet, stabilized by water exclusion interactions between the oil phase and the hydrophobic crystalline plane of CNCs [91]. Cellulose nanomaterials have also been used to stabilize inverse water-in-oil emulsions. In order to achieve this, surface modification with hydrophobic groups was performed. The hydrophobic modifications proposed included silylation, which showed an increased emulsion stability with increasing degree of substitution, [92] acetylation, [93] acylation, [94] quaternary ammonium salts, [95] or lauroyl chloride, which was used to stabilize double (oil-in-water-in-oil) emulsions [96]. Cellulose nanocrystals have been also shown to stabilize water-in-water emulsions formed by dextran and poly(ethyleneoxide) [97]. Chitin nanomaterials are structurally related to cellulose nanomaterials, and possess similar amphiphilic behavior, which makes them good candidate as stabilizers for oil-in-water Pickering emulsion [98,99]. Starch possess also amphiphilic properties, and starch microgranules were used to stabilize oil-in-water Pickering emulsions, [100] however showing limited stabilization with respect to starch nanocrystals, [101,102] and nanospheres [103]. This may be associated with the lower size and the higher aspect ratio of the platelet-like structure. Hydrophobic modification of the starch granules resulted in more stable oil-in-water emulsions, [104] and were shown to form stable multiple (*w*/*o*/*w*) Pickering emulsions [105]. As a general route, the microcapsule preparation involved the stabilization of the emulsion in a first step, followed by the reinforcement of the interface to yield solid particles. This method results in liquid-filled microcapsules, which can be used to encapsulate oils. The soft template can also be removed by drying to yield hollow microcapsules. In order to have a better control over the droplet size, and to decrease emulsion polydispersity, microfluidic devices could be used to generate emulsions [106,107].

As a coating method, the layer-by-layer approach (LbL) is particularly interesting for microcapsules preparation, as it allows to control the size, shape, composition, and to insert functional molecules [108]. It can be applied to both soft and hard templates. This method has been extensively applied to prepare polymeric polysaccharide microcapsules [17]. All types of interactions could be used. Systems of polymeric polysaccharide-based microcapsules stabilized by electrostatic interaction, hydrogen bonding, covalent crosslinking, ionic cross-linking, and host–guest interactions, have been reported [12]. Interactions between opposite charges are of particular interest for polysaccharide nanomaterials, as binding can be controlled by the degree of substitution, and their surface chemistry could be further exploited for controlled attachment of drugs or other species [109,110]. The layer-by-layer approach has been widely used to prepare nanocellulose-based films in combination with oppositely charged polyelectrolytes, [111] or with other cell wall polysaccharides such as xyloglucan [112]. Examples of chitin multilayers films based on the LbL approach have been also reported [112,113]. A specific type of electrostatic interaction used to prepare polysaccharide microcapsules is ionic cross-linking, where small ions or polyions react with charged polysaccharides, forming ionic bridges along the polysaccharide chains [12]. This method has been applied to form chitosan microcapsules, for example in combination with polyanion polyphosphate as ionic cross-linkers [114,115]. Anionic polysaccharides such as alginate have been also commonly used to prepare microcapsules by ionic cross-linking, for example by treating an aqueous solution of alginate with a CaCl_2_ solution [116]. Chemical cross-linking reactions have been also commonly employed to prepare polysaccharide microcapsules. The main advantage of this method is the more robust coating, stabilized by covalent bonds. In particular, the most common strategies for preparation of polysaccharides microcapsules involved Schiff base bonding and amine-carboxylic acid bonding [12].

## 4. Microcapsules Based on Polysaccharide Nanomaterials

Although many examples of soluble polymeric polysaccharide microcapsules have been reported, only recently these methods were applied to polysaccharide nanomaterials. In this section, we will describe and review the works published in the literature, focusing on the main preparation methods proposed, describing the various structures created and the main interactions involved.

### 4.1. Ion Cross-Linked Polysaccharide Microcapsules Reinforced with Polysaccharide Nanomaterials

**Figure 2:**
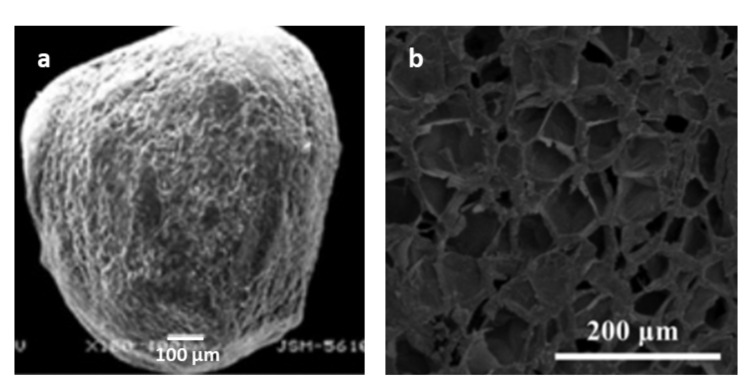
(**a**) SEM image of alginate-cellulose nanocrystals (CNC) microcapsules. Reprinted with permission from [117] Copyright © 2011 Published by Elsevier Ltd. (**b**) Surface morphology showing the porous nature. Reprinted with permission from [125] Copyright © 2019 Published by Elsevier Ltd.

To our knowledge, the first work where polysaccharide nanoparticles were used to prepare microcapsules was published on 2011. The authors incorporated cellulose, chitin and starch nanocrystals into alginate microspheres, in order to improve mechanical strength and to regulate drug release behavior [117]. Various other systems of alginate microcapsules reinforced by polysaccharides nanomaterials have been presented in literature and were used to encapsulate various molecules. An overview of the reported works is shown in Table 1. Negatively charged polysaccharide nanocrystals can participate in the crosslinking between calcium ions and alginate [118]. Additional interactions can be associated to a hydrogen bond network between the carboxylic groups of alginate and the hydroxyl surface groups of polysaccharide nanocrystals [119]. The incorporation of polysaccharide nanocrystals (in a 1:1 weight ratio) improved the mechanical properties of the alginate microcapsules. In particular, the incorporation of rod-shaped cellulose and chitin nanocrystals resulted in stronger microcapsules, compared to platelet-shaped starch nanoparticles. This behavior was linked to the higher aspect ratio of the rod-like shapes and to weaker interactions of alginate with starch nanocrystals due to their self-aggregation properties. Formation of a more rigid network decreased the porosity, resulting in improved drug encapsulation efficiency and a better control over drug release behavior. The alginate/nano-polysaccharide microcapsules presented limited swelling behavior and drug release at pH 1, compared to neutral pH [117]. Lemahieu et al. prepared alginate microcapsules coated with cellulose nanocrystals and nanofibers, and showed that they were strong enough to keep their integrity after extrusion [120]. Other works have also highlighted the ability of cellulose nanocrystals to improve mechanical stability of alginate composites, [121] and showed that reinforced alginate-CNC microcapsules were strong enough to stabilize the encapsulation of bacteria. The microcapsules presented high swelling properties at pH 7, and also at acidic pH [122]. The difference with the previous paper, where the particles did not swell at acidic pH, [117] is linked to the lower amount of cellulose nanocrystals used in this work (13% CNC), compared to the previously mentioned article (50% CNC), demonstrating that incorporation of CNC decrease swelling properties of alginate microcapsules. Alginate-CNC microcapsules were also used to encapsulate nisin, which is a food preservative, and thyme oil [123,124]. Yan et al. were able to encapsulate a CNC stabilized water/dichloromethane emulsion previously loaded with a hydrophobic drug in alginate microcapsules [125]. Other groups have prepared alginate-CNC systems for removal of organic and inorganic pollutants from water by encapsulation, which take the advantage of the easy separation method of the solid capsules [126,127,128]. The incorporation of carboxylated cellulose nanocrystals can be particularly interesting for such application, as interactions with cationic species are pH dependent and can be easily regenerated by lowering the pH [129]. Taken together, the microcapsules prepared combining alginate with polysaccharide nanomaterials showed a similar structure, with a large diameter of several hundred µm, and a porous morphology, as shown in Figure 2. In a recent work, negatively charged cellulose nanocrystals were used as polyion cross-linker for preparation of chitosan microcapsules for encapsulation of anthocyanins. The authors highlighted the potential of CNC as ionic cross-linking agent, as it also acted as filler for the chitosan matrix, generating more rigid microcapsules with improved encapsulation efficiency and stability, compared to tripolyphosphate (TPP) microcapsules [130].

### 4.2. Biomimetic Polysaccharide Nanomaterials Microcapsules Stabilized by Electrostatic and H-Bond Interactions

**Figure 3:**
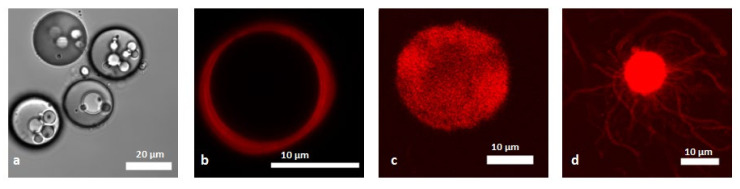
(**a**) **Confocal Laser Scanning Microscopy** (CLSM) images of microcapsules with lipid interior. (**b**) CLSM image of a single plantosome containing Rh-DOPE (red) in the lipid phase. (**c**) CLSM images of plantosome expanded in a 100mM NaCl solution after 1 h at pH 8.6. (**d**) CLSM images of a lipid tubular protrusions from expanded plantosomes. Adapted from ref [137].

Although alginate microcapsules reinforced with polysaccharide nanomaterials are promising systems for loading and release of drugs, food, or other active components, and also for water cleaning, the resulting particles were big compared to natural architectures such as the plant cell wall. In fact, the particle size ranged from several hundred microns to more than 1 mm, while the dimension of natural plant cells is around 10–100 μm [131]. The preparation of microcapsules of even smaller size are also of great interest for drug delivery applications, as they may enter the intestinal mucosa, enabling better contact with the site of adsorption and better stability [132]. Several systems of microcapsules inspired by nature, using polysaccharide nanomaterials as coating, have been reported, employing electrostatic and H-bond interactions to stabilize the structure. A list of the reported work is shown in Table 2. A popular strategy to prepare smaller microcapsules prepared by only natural components is by coating hard templates of smaller size in a LbL approach, followed by removal of the template. A first system of CNC-based microcapsules with diameter of few micrometers was reported by Mohanta et al., which used the layer-by-layer approach on a melamine formaldehyde sacrificial template [133]. Chitosan was used as the second component, as it interacts with CNC by electrostatic interactions. The sacrificial template used was a synthetic material, but was dissolved in 0.1 M hydrochloric acid, resulting to water-filled hollow microcapsules composed entirely by polysaccharides. Such particles allowed drug molecules with good water solubility to be loaded in the aqueous interior by diffusion, while hydrophobic drugs could be encapsulated by the preparation of drug conjugates with CNCs. At least five bilayers were required to prepare stable microcapsules. Ye et al. used SiO_2_ microspheres as sacrificial template to prepare microcapsules of around 4 μm using layer-by-layer approach, by employing cellulose nanocrystals as the main component [134]. Heat was used to facilitate the formation of hydrogen bonds between cellulose nanocrystals layers to stabilize the multilayer complex. The authors succeeded in adding up to 18 layers and showed low growth rate as a function of the number of layers, compared to other LbL systems based on traditional polyelectrolytes, due to the repulsion forces between layers of the negatively charged CNCs. Harsh treatment was required to obtain hollow microcapsules, as dissolution of the SiO_2_ core was performed in a 1 M HF/4 M NH_4_F. The authors studied the permeability of the hollow microcapsules in water, showing porosity larger than 100 nm for the three-layer system. The permeability decreased with increasing number of layers, but the microcapsules formed by 18 layers were still permeable to particles of 30 nm diameter. The encapsulation and release properties of these microcapsules could be controlled by external pH. At an acidic pH of 1.5, the capsules shell opened, allowing polystyrene nanoparticles, which were used to investigate porosity, to penetrate inside the microcapsules. Adjusting the pH to 7.5 the particle shell closed enabling loading of the particles, which were released by decreasing pH. It should be mentioned that a synthetic cationic polymer (PEI) was required as a first layer to facilitate the adsorption of the CNCs on the template, making the resulting microcapsules not fully biobased. However, this method is still interesting, as the synthetic polymer could be replaced by a natural polyelectrolyte. Paulraj et al. published a series of articles where spherical calcium carbonate crystals were used as hard template to prepare microcapsules formed by only the polysaccharides that constitute the plant cell wall (cellulose, pectin, and xyloglucan) [135,136,137]. Cellulose nanofibers modified with a cationic surface group were employed. In a first presented work, a layer-by-layer approach was used with anionic apple pectin (AP) as the first layer, and cellulose nanofibers as the second component [135]. 5 AP/CNF bilayers were added on a CaCO_3_ sacrificial template, followed by an additional AP layer and a final xyloglucan (XgG) layer, which could crosslink the other components, resulting in further stabilization of the structure. Adding more than five bilayers caused significant aggregation, which destabilizes the capsules structure. In fact, cellulose nanomaterials and apple pectin can cross-link particles, which represents the main difficulty of using the layer-by-layer approach to create microcapsules. The particles prepared were further stabilized by dissolving the calcium carbonate core using citric acid, which was adsorbed on the multilayer structure, making it more compact, decreasing porosity and swelling. The capsules diameter was of around 16 μm, comparable to the size of the sacrificial template. The wall size was 60 nm, larger than the previously reported capsules, furnishing higher mechanical stability. Upon drying, the microcapsules collapsed, exhibiting the formation of random wrinkles due to the polymers adsorbed on the capsules wall. The permeability of the particles prepared was dependent on the ionic strength, as the microcapsules prepared were permeable to 70 kDa dextran in presence of 10 mM NaCl, and non-permeable in water. When NaCl was removed by washing with water, dextran remained trapped inside the capsules, and was released by re-adding salt. In the same work, the authors attempted to insert xyloglucan between CNF and AP, but without success, [135] since pectin and xyloglucan compete for binding to CNF, [138] preventing the formation of a multilayer structure. In a second reported work from the same group, xyloglucan was inserted in the multilayer structure creating a 3 polymer system in the sequence (CNF/XyG/CNF/AP)_2_CNF/XyG [136]. The particles had a thinner wall compared to the CNF/AP microcapsules presented in the previous work (around 20 nm). Another difference with the particles presented in their previous work was the permeability. The particles were permeable to 70 kDa dextran in water. The addition of salt decreased the pore size, and at 250 mM NaCl the microcapsules wall closed entrapping the dextran molecule inside. Washing off the salt enabled the release of dextran. The authors associate the differences with the previous work (where the on/off switch is reversed [135]) with the absence of citrate on the capsules wall, as 250 mM EDTA was used to dissolve the sacrificial template. In the same publication, the authors prepared microcapsules by multilayer adsorption of CNF and XyG (up to 5 bilayers) [136]. These microcapsules were less stable than the (CNF/XyG/CNF/AP)_2_CNF/XyG capsules, due to the higher tendency of aggregation and due to the lower amount of polymer on the capsule wall (wall thickness was of around 10 nm), as pectin acted as a filler material. In fact (CNF/XyG)_5_ microcapsules partially lost their spherical shape after core removal, and the microcapsules were not used to study permeability. (CNF/XyG/CNF/AP)_2_CNF particles presented large pores also in a biological buffer (DPBS). Such microcapsules were suitable for enzyme immobilization (glucose oxydase), and provided a favorable and flexible microenvironment that preserved the enzyme activity [139]. In a recent work, a soft template was used to prepare microcapsules system with the highest resemblance with the plant cell, which the authors defined as plantosomes.[137] An overview is presented in Figure 3. The authors used a Pickering emulsion stabilized by CNF as template, consisting of an oil-in-water emulsion stabilized by cellulose nanofibers where the oil phase consisted of oleic acid/oleate dissolved in chloroform. Oil-filled microcapsules (see Figure 3a) were prepared by adding a layer of pectin as filler material, and then evaporating the chloroform. Adding a small number of phospholipids in the oil phase resulted in water-filled microcapsules (see Figure 3b). The microcapsule structure expanded at higher pH, due to the pH dependent phase behavior of oleic oil/oleate in water, [140] and formed lipid vesicles in the capsule’s interior (see Figure 3c). In some cases, thin tubular lipid protrusions were observed, formed to release the pressure caused by the crowded lipid interior (see Figure 3d) [137]. Kaufman et al. reported the preparation of water-filled microcapsules composed of carboxylated CNF, using a water-in-toluene emulsion as soft template. The emulsion droplets transformed into microcapsules by a complex formation between the negatively charged cellulose nanofibers and a random cationic copolymer previously dissolved in the oil phase. The microcapsules prepared were significantly larger than the previous examples (with diameter of around 300 μm, and wall size of 12 μm), and required a microfluidic device to reduce emulsion droplets and therefore capsules size [141]. Song et al. used bacterial cellulose to prepare microcapsules of various sizes, using an oil-in-water emulsion droplet as soft template. A bacterial culture was used in the water phase (which consisted in a fermentation medium) and allowed the production of cellulose fibers around the oil droplets (castor oil was used as oil phase) created in a microfluidic device. The size of the microcapsules was set by the initial droplet size, and ranged from 20 µm to several mm [142]. Li et al. used negatively charged cellulose nanofibers to stabilize paraffin-in-water emulsions, enabling paraffin encapsulation. Ion-assisted gelation was further performed to prepare an hydrogel stabilized by microcapsules with energy storage properties [143]. Levin et al. reported a preparation route of bioinspired microcapsules formed by self-organization of CNC in a water-in-oil emulsion, where water droplets were generated by a microfluidic device. An emulsifier was required to prevent coalescence. To improve stability of microcapsules, the authors used a mixture of cellulose nanocrystals modified with aldehyde and hydrazone group, which formed cross-linked structures that reinforced the capsule shell. The size of the microcapsules could be tuned by varying the starting CNC concentration. The authors also studied the self-organization of the nanocrystals at the interface, showing that droplets formed by unmodified (sulfated) CNC formed chiral-nematic domains upon drying, while drops stabilized by physically cross-linked CNC did not exhibit self-assembly properties. After rehydration, the microcapsules showed radially oriented structures, but lacked a chiral nematic texture [144]. Jativa et al. showed that aqueous suspensions of carboxylated CNC were able to self-assemble in a toluene/ethanol during droplet dissolution, and that the capsules obtained by solvent evaporation, which were larger than previous examples (≈ 300–800 µm), were able to retain the ordered structure at low ethanol concentration (< 5%) [145]. Smaller CNC microcapsules were obtained generating water droplets in a hexadecane phase using a microfluidic device, and also showed a preservation of the chiral nematic phase upon drying [146]. The self-assembly of starch nanocrystals in an oil(n-hexane)-in-water interface were also recently exploited to form robust microcapsules, which were stable after freeze drying [147]. A work has been reported where the ability of chitin nanocrystals to stabilize the Pickering emulsion was exploited to encapsulate paraffin. The ChiNC-stabilized paraffin-in-water emulsion was homogenously dispersed in a starch matrix, to form a composite film by solvent casting. The process was performed at different temperatures, to encapsulate paraffin in solid and liquid state. In both cases, the microcapsules presented high stability, and remained intact after film processing. Enzymatic digestion of starch allowed the liquid droplets to be released [148].

**Figure 4:**
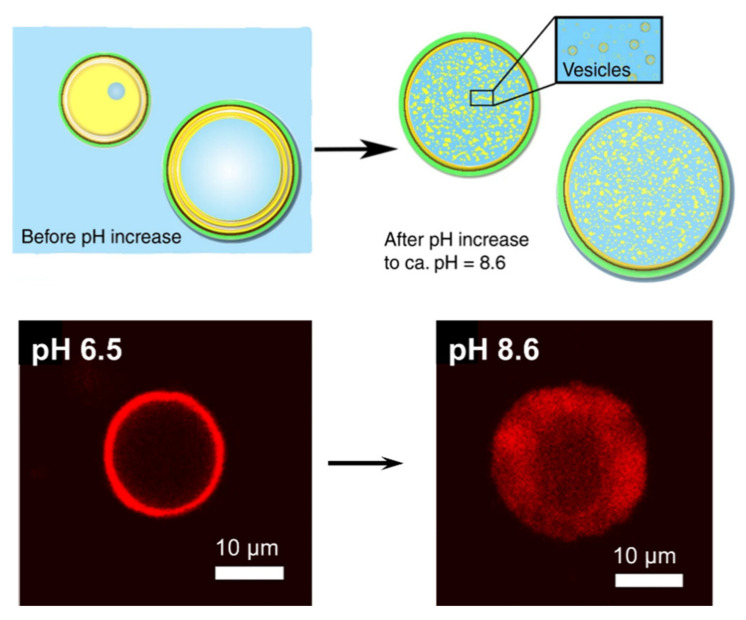
Proposed mechanism for the formation of vesicles in the microcapsule interior and CLSM images at pH 6.5 and pH 8.6. Adapted from ref. [137].

The use of electrostatic and hydrogen bonds interactions to stabilize coating is of particular interest for preparation of microcapsules systems inspired by natural architecture. Such systems are particularly interesting to prepare microcapsules with stimuli-responsive properties, as such interactions are reversible and could be controlled by varying experimental conditions (such as ionic strength, pH, or temperature). However, only few works have been presented in literature. The reported microcapsules varied in composition. In fact, polysaccharide materials were combined with different components (see Table 2). Even the cellulose nanomaterials (which were used in most of the reported works) presented significant structural differences, such as size and charge. The resultant microcapsules showed different stimuli-responsive properties, such as tuned porosity and permeability, and mechanical and thermal properties. Figure 4 illustrates an example of pH-responsive properties. Microcapsules composed of an outer shell of CNF and pectins, containing a thin layer of lipids beneath, showed a pH-responsive formation of vesicles in their interior that change microcapsule dimensions.

In addition, the systems presented were stable in water but collapsed upon drying. More work is therefore required to prepare bio-inspired nano-polysaccharide-based microcapsules with high stability using electrostatic and H-bonds interactions to stabilize the coating, and to fully exploit their stimuli-responsive properties.

### 4.3. Microcapsules Prepared by In Situ Core Formation Stabilized by Polysaccharide Nanomaterials

**Figure 5:**
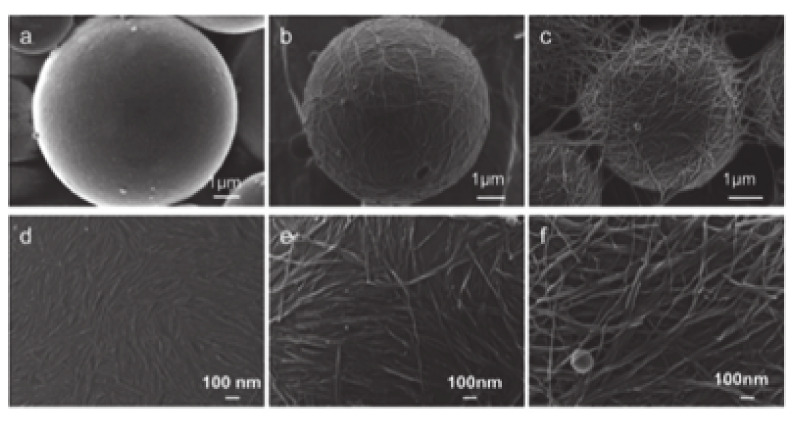
SEM images of polymerized styrene–water emulsions stabilized by cellulose nanomaterials of different sizes. (**a**,**d**) Cellulose nanocrystals from cotton, ≈ 200 nm in length. (**b**,**e**) Bacterial cellulose nanocrystals, ≈ 900 nm in length. (**c**,**f**) cellulose nanofibers from cladophora, ≈ 4 µm in length. Reproduced from Ref [149] with permission from The Royal Society of Chemistry.

In order to reinforce the microcapsule coating, researchers have used covalent bonds to stabilize the structures. Several works have reported the polymerization in a styrene-in-water emulsion stabilized by cellulose nanomaterials to form coated polystyrene microspheres. Such a system was mostly used for studying emulsion behavior, as the solid beads formed are robust, and allows visualization by scanning electron microscopy [89]. Nanocrystals of smaller size are more convenient because a more dense organization on the polymer surface is created, while longer CNC formed an interconnected network [149]. Larger cellulose nanofibers resulted to larger structures with less surface coverage, connected by a strong interparticle network [150,151]. A better overview of the size range and of the structures of the resulting particles is shown in Figure 5.

**Figure 6:**
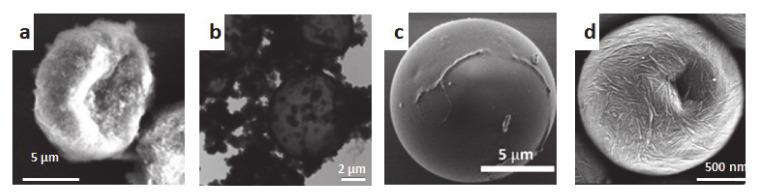
Examples of microcapsules stabilized by covalent bonds. (**a**) SEM imaging of a CNC−CoFe_2_O_4_ microcapsule prepared by dissolution of the polystyrene core. Reprinted with permission from [153] Copyright (2014) American Chemical Society. (**b**) TEM images of polydopamine microcapsules templated by CNC stabilized Pickering emulsions. Reprinted with permission from [161] Copyright © 2019 Published by Elsevier Ltd. (**c**) SEM image of cross-linked Cin-CNC/silica colloidosomes. Reprinted with permission from [162]. Copyright (2018) American Chemical Society. (**d**) SEM image showing a microcapsule structure obtained by cross-linking with isophorone diisocyanate. Reprinted with permission from [163]. Copyright (2014) American Chemical Society.

Varying the surface charge of CNCs showed no influence on the microcapsule size, provided that enough salt is added to partially suppress the electrostatic repulsion between nanocrystals [152]. However, at lower surface charge, the particles presented a thicker CNC coating [91]. Loading the CNCs with magnetic responsive nanoparticles did not influence the stability of the emulsion, and allowed preparation of microspheres with magnetic properties [153]. These systems can be used as platform for multilayer adsorption, which would allow modulating the particles charge and provide further stabilization [152]. Surface modification of CNCs, using a pH-responsive amide group at the reducing end, improved the stability of the emulsion, and were used to prepare CNC-coated polystyrene microbeads with amino-rich surface [154]. Werner et al. used acetylated CNCs to coat polystyrene, and obtained two populations of particles, one with diameter in the micrometer range, and a second with nanometer size [155]. Smaller CNC-coated polystyrene particles with diameter of few hundreds nanometers were obtained using cellulose nanocrystals hydrophobically modified with alkyl-amines [156]. A related strategy can be employed with other polymeric systems [157,158]. It should be pointed out that, in these works, the particles internal morphology was not studied, thus the term microbeads or microspheres was mostly used by the authors. However, these systems represent an alternative strategy for the preparation of latex particles and polymeric microcapsules without using surfactants, which are known to have negative effects on the particles properties, such as the water resistance of the coating [159]. Nanocellulose-coated polymeric microspheres represent an interesting template for the preparation of hollow nano-polysaccharide microcapsules, as the polymeric core can be dissolved. An overview of nanopolysaccharide microcapsules, where the presence of a hollow interior has been shown, is presented in Figure 6. For example, Nypelo et al. used acetone to dissolve the polystyrene core (see Figure 6a) [153]. Zhang et al. showed that polymerization of styrene and other hydrophobic monomers, performed in a water-in-toluene Pickering emulsion stabilized by CNC, formed polymeric microcapsules with a hollow interior, resulting from voids created by toluene removal [160]. Hydrophilic monomers were used to prepare microcapsules by polymerization, using an inverse water-in-oil emulsion stabilized by hydrophobic (cinnamate modified) CNCs [160]. However, the presented microcapsules were fully polymeric. The same hydrophobic modification was used to prepare robust polydopamine microcapsules, which could be used to efficiently encapsulate essential oil and pesticides (see Figure 6b), [161] and as a template for the polymerization of orthosilicates (the authors used either tetraethyl and tetrabutyl orthosilicate as monomers), to form CNC/silica colloidosomes that were able to encapsulate DNA (see Figure 6c) [162]. A different strategy was used by Svegan et al., which used a water-in-hexadecane emulsion stabilized by a mixture of CNC and CNF as template. A polyaddition reaction with isophorone diisocyanate was used to prepare nanocellulose-based microcapsules. Such reaction created a cross-linked network structure around the oil droplets, resulting in liquid core microcapsules of around 1 μm, which presented high mechanical stability. The obtained microcapsules contained only 17% of nanocellulose, while the main structural component consisted in a polyurea/polyurethane matrix, which was the result of the high amount of crosslinking required to fully cover the oil droplets (see Figure 6d) [163]. In order to increase nanocellulose content, and to prepare microcapsules with oxygen barrier properties, these capsules were blended with a suspension of carboxylated cellulose nanofibers and passed by a hydrophobic membrane. The microcapsules obtained by solvent evaporation contained a higher amount of nanocellulose, presented a bigger size (of around 8 μm), and a highly folded structure [164]. In another reported work, cross-linked CNC/CNF microcapsules were coated with silicon oxide, which formed silicon nanospheres on the coating, resulting in broccoli-shaped microcapsules of around 1–2 μm. The capsules presented superamphiphobic properties, being both highly hydrophobic and oleophobic [165]. In a recent work, a series of microcapsules with paraffin as the core material, and melamine–urea–formaldehyde polymer as coating, were manufactured with different contents of CNCs via in situ polymerization [166].

To our knowledge, very few examples have been presented using Pickering emulsions stabilized by other polysaccharide nanoparticles as template to prepare cross-linked microcapsules. For instance, chitin nanocrystals were also used as a template to prepare polystyrene microspheres, showing a similar structure and characteristics of the polystyrene particles coated with CNC [167]. Preparation of SNC-coated polymeric microspheres were obtained from cross-linking of silicone oil in an oil-in-water emulsion, [102] and from in situ polymerization of butyl methacrylate with size lower than 1 µm [168]. However, in these works, the polymeric core was not dissolved. In another reported work, a colloidal water suspension of chitosan nanoparticles was used to stabilize a dichloromethane-in-water emulsion, which was used as template to form microcapsules in combination with poly(lactic-co-glycolic acid), which were cross-linked to increase stability [169].

## 5. Conclusions and Perspectives

A variety of microcapsules have been presented in the literature, using different interactions to stabilize the coating, and different hard and soft templates. Most of the reported works have used nanocellulose as polysaccharide nanomaterial, but the same principles could be applied to other polysaccharides, in particular the use of chitin nanoparticles could be interesting for a better comparison, due to their structural similarity with nanocellulose. Among the reported structures, the alginate-CNC microcapsules had higher stability, and represent a promising system for delivery of drugs or food, and a variety of active components has been encapsulated. A limitation of these systems is that microcapsules presented were too large, and efforts should be made to achieve a better control over the size. Microcapsules stabilized by reversible interactions, such as electrostatic and H-bonds, represents the most interesting system, as they are particularly promising for development of microcapsules with stimuli-responsive properties. However, more work is required in order to fully exploit these properties. Most of the systems reported were formed by only natural compounds, and in some cases, microcapsules with high resemblance with the plant cell wall have been reported. The stability of these structures is the main limitation, as most of the microcapsules were stable in suspension, but collapsed upon drying. The nature and extent of interactions between the microcapsule components determine its stability. Therefore, the control of supramolecular interactions is the key point in microcapsule fabrication. For this purpose, the use of the surface chemistry of nanocellulose could be of particular interest, as it allows to modulate the surface charge and the specific interactions on nanocellulose surfaces and can be exploited to prepare stronger coatings. We think that associating the structure formation with the thermodynamic measurement of the interaction between components could be of great importance, as it will give better information on the nature and the strength of interaction within the coating components, which can be used to prepare more stable microcapsules. The surface chemistry could also be used to tailor specific interactions between the coating material and the encapsulated species, which could be of great interest to achieve a better control of the load-release properties, compared to the few systems presented, which used a diffusion mechanism to encapsulate species.

Some attempts to use of covalent bonds to increase structure stability have been also presented, but the resulting microcapsules were mostly composed by synthetic materials, and the development of more green pathways is required to prepare systems that could be exploited in delivery of active compounds.

## Figures and Tables

**Table 1:** Reported alginate/nanocrystals microcapsules for encapsulation of active species.

Nanomaterial	Encapsulated Specie	Properties Studied	Ref
CNC ChiNC SNC	Theophilline	Swelling in water - loading and release	[117]


CNC	Probiotics	swelling and dissolution under simulated gastrointestinal conditions	[122]
CNC	Nisin	loading and release	[123]
CNC	Thyme oil	loading and release	[124]
Bacterial CNC	α-Calcidol	loading and release	[125]
CNC	Methylene blue	Thermodynamics of interactions	[126]
CNC (containing lignin)	Methylene blue	Thermodynamics of interactions	[128]
CNC	Pb^2+^	Thermodynamics of interactions	[127]

**Table 2:** Microcapsules stabilized by polysaccharide nanomaterials stabilized by electrostatic and H-bonds interactions.

Template Used	Coating Components	Encapsulated Species	Properties Studied	Size (µm)	Ref
Melamine formaldeyde	(chitosan /CNC)_n_	Doxorubicin hydrochloride	Loading and release	3.3–3.5	[133]
SiO_2_	PEI(CNC)_n_	Polystyrene beads	Permeability in water	3.8 ± 0.5	[134]
CaCO_3_	CNF/AP/ XyG (AP/CNF)_5_AP/CNF	Dextran	Permeability in water	16 ± 4	[135]
CaCO_3_	(CNF-XyG)_5_ (CNF/XyG/CNF/AP)_2_/CNF/XG	Dextran BSA	Permeability in water/NaCl and cell growth media	16 ± 4	[136]
CaCO_3_	(CNF/XyG/CNF/AP)_2_CNF	Dextran	Permeability in biological buffer - GOx loading efficiency – enzyme activity	12 ± 2	[139]
Oil-in-water emulsion	CNF/Pectin		Porosity – pH dependent structure and expansion	27	[137]
Water-in-oil emulsion	CNC/cationic polymer		Mechanical properties	303 ± 3.4	[141]
Oil-in-water emulsion	Bacterial cellulose		Porosity, mechanical properties	from 100 to few cm	[142]
Oil-in-water emulsion	CNF	Paraffin	Mechanical and thermal properties	5–10	[143]
Oil-in-water emulsion	ChiNC	Paraffin		2–5	[148]
Water-in-oil	CNC(SO_4_) CNC(aldehyde)-CNC (hydrazone)		Swelling, porosity, self-assembly	> 300	[144]
Water-in- toluene/ethanol	CNC(COOH)		Self-assembly	300–800	[145]
Water-in-hexadecane	CNC(SO_4_)		Self-assembly	≈ 20	[146]
n-Heptane-in-water	SNC	enzyme	Catalytic activity	5–30	[147]
